# Light Microscopy and Proteomic Patterns of Ovulation in Cervical Mucus

**DOI:** 10.3390/life12111815

**Published:** 2022-11-07

**Authors:** Yolanda Fernandez-Hermida, Federica Vincenzoni, Domenico Milardi, Anna Laura Astorri, Andrea Urbani, Giuseppe Grande, Rafael Azagra

**Affiliations:** 1 Department of Medicine, Faculty of Medicine, Internacional University of Catalonia, 08195 Sant Cugat del Vallés, Spain; 2 Dipartimento di Scienze Biotecnologiche di Base, Cliniche Intensivologiche e Perioperatorie, Università Cattolica del Sacro Cuore, 00168 Rome, Italy; 3 Fondazione Policlinico Universitario “A. Gemelli” IRCCS, 00168 Rome, Italy; 4 International Scientific Institute “Paul VI”, 00168 Rome, Italy; 5 Division of Endocrinology, Fondazione Policlinico “A. Gemelli” IRCSS, 00185 Rome, Italy; 6 Unit of Andrology and Reproductive Medicine, Department of Medicine, University of Padova, 35121 Padova, Italy; 7 Health Center Badia del Valles, Institut Català de la Salut, 08214 Badia del Vallés, Spain; 8 GROIMAP Research Group, Research Support Unit Metropolitana Nord, Instituto Universitario IDIAP Jordi Gol, 08290 Cerdanyola del Vallés, Spain; 9 Fundació Privada PRECIOSA per la Investigació, 0821 Barberá del Vallés, Spain

**Keywords:** infertility, reproduction, biomarker, ovulation, cervical mucus, fertile window, proteomics

## Abstract

There is an increasing number of couples interested in identifying the fertile window for the purpose of conceiving. From what has been published so far, it can be concluded that there are no reliable methods to predict ovulation, and, therefore, to predict the fertile window. Proteins of the cervical mucus (CM) could behave as biomarkers to allow the early and precise identification of ovulation. CM samples were collected from the lumen of the cervical canal from women of reproductive age, on three different days of the same menstrual cycle. Samples were first analyzed and classified by light microscopy. High-resolution mass spectrometry and bioinformatic analysis were performed afterwards to determine the in vivo changes of CM protein composition. CM underwent cyclical changes in its biophysical composition, which were evidenced by changes in the crystallographic patterns observed under the light microscope. The proteomic analysis revealed changes in the protein composition of CM along the cycle. Twenty-five out of the forty-eight total proteins identified could become potential biomarkers of ovulation. The coordinated changes in the composition of the CM around the time of ovulation could be happening to specifically grant access to a foreign body, such as the sperm might be.

## 1. Introduction

### 1.1. Menstrual Cycle and Fertile Window

The menstrual cycle begins on the first day of menstruation, and ends on the last day before the next menstruation. Ovulation generally occurs only once in each menstrual cycle. The ovum dies in 10–24 h if it is not fecundated [1]. In each menstrual cycle, there is a limited period of time, during which the woman can conceive. This period is known as the “Fertile Window” and includes the days preceding ovulation and the day of ovulation itself [2,3,4]. Studies suggest that the “fertility window” begins 5–6 days before ovulation and ends on the day of ovulation [5].

The clinical guidelines establish a mean duration of the menstrual cycle of 28 days; however, there is great variability in the duration of the cycle between women and within the same woman [6,7,8,9]. A study of 612,613 cycles revealed that only 13% of menstrual cycles were 28 days long [10]. In about 30% of women, the fertile window is completely within the days of the menstrual cycle identified by clinical guidelines; that is, between days 10 and 17 [6]. Likewise, intracycle variability of more than 7 days was observed in 42.5% of women [7]. This variability in the duration of the cycle is mainly attributed to the follicular phase, which is variable in duration [9]. The fertile window is, therefore, highly unpredictable even when cycles are regular [6].

Since the 1920s, several biomarkers have been investigated for the purpose of identifying ovulation in advance. Over the past several years, the interest in determining fertility has greatly increased among the general population. In the United States of America, for example, it has been estimated that more than 7 million women have sought professional fertility treatment, and nearly 450,000 of those women sought medical attention for advice on issues such as detecting the fertile window and optimizing the timing of sexual intercourse [1,11]. Currently, there are more than 500 fertility related mobile applications [12]. Self-identification of the fertile window is either used to postpone pregnancy [13,14,15,16] or to achieve pregnancy [17,18].

From what has been published so far, it can be concluded that there are no reliable methods to predict ovulation and, therefore, to predict the fertile window [6].

### 1.2. Cervical Mucus

Cervical mucus (CM) is a viscous fluid produced by the secretory cells of the cervical crypts of the uterus [19,20]. The CM undergoes modifications throughout the menstrual cycle that make it have different biochemical and biophysical characteristics.

#### 1.2.1. Biophysics of the Cervical Mucus

Professor E. Odeblad has made an extraordinary contribution to the knowledge of the physiology of CM. He has identified four main different types of CM: S mucus (string, sperm-conducting), L mucus (loaf, locking in low-quality spermatozoa), P mucus (peak, because it has its maximum secretion on the peak day [21]), and G mucus (gestagenic). These types of CM have been studied using light microscopy and nuclear magnetic resonance techniques [22,23,24].

#### 1.2.2. Biochemistry of the Cervical Mucus

From a biochemical point of view, CM consists of two main fractions: an aqueous phase of low molecular weight, the cervical plasma, and a viscoelastic gel phase matrix composed of a polymer of glycoprotein with high molecular weight (mucin) [17].

The aqueous phase is mainly composed of water (90–98%). The aqueous phase also contains electrolytes (Na^+^ and Cl^−^) and other soluble components, such as enzymes, trace metals, serum proteins, and immunoglobulins of local origin [25,26].

MUC5B is the main gel-forming mucin of the endocervical epithelium [27].

The chemical composition of the CM, its physical characteristics, and the volume of secretion change cyclically throughout the menstrual cycle. Total CM proteins decline at the time of ovulation [28]. Most of the proteins in CM show a cyclical pattern [29], which implies a decrease in certain soluble proteins and the appearance of others from 3 to 5 days prior to ovulation [30].

### 1.3. Cervical Mucus: Functions

Among other relevant functions, the cyclical variability of CM provides information on the fertility status of women throughout the cycle [31,32]. There is evidence that CM is a crucial element for the identification of the time of ovulation [33,34,35]. There are numerous indications that CM is used as an indirect element for estimating the time of ovulation, both for physicians and women using fertility awareness-based methods [36,37,38].

### 1.4. Cervical Mucus Proteomics in Fertile Women

The research in human physiology, particularly in biological fluids, has benefited from the rapid development of proteomic technology. Thanks to this efficient tool, it is now possible to identify new potential biomarkers for prognosis, therapy, and diagnosis, as well as the comprehensive characterization of the proteomic composition in different clinical aspects, including reproduction [39]. The proteomic composition of CM, cervical vaginal fluid, endometrial fluid, and MC plugs has been investigated in some studies [40,41,42,43,44,45,46,47,48,49,50,51,52,53,54,55]. The proteomic composition of CM is probably less complex than urine and plasma, but how the CM proteome undergoes cyclical changes and how many proteins CM contains are still uncertain [56]. Modifications in the CM proteome during the different phases of the menstrual cycle were analyzed for the first time in 2015 by Grande et al. using high-resolution mass spectrometry, implemented using quantitative tools. The proteomic approach revealed differences in the expression of various CM proteins during the menstrual cycle [56].

The present work aims to contribute to the knowledge of the properties and physicochemical functions of CM, including its important potential in the role as a clinical marker of female fertility.

## 2. Materials and Methods

Six 25-to-30-year-old fertile women with no history of fertility problems and a history of regular menstrual cycles were enrolled. The volunteers gave informed consent according to the guidelines of the Declaration of Helsinki. The individuals made the commitment to maintain sexual abstinence during the study.

CM samples (N = 3 per patient) were obtained by gentle aspiration of the cervical canal lumen using an intrauterine insemination catheter (Gynétics Medical Products, Achel, Belgium). Samples were collected on days 7, 12, and 18 of the same menstrual cycle. A portion of the CM sample was placed on a slide (Waldemar Knittel, Braunschweig, Germany) and radiated in all directions by using a needle according to the spread-out technique described by Professor Odeblad in 1995. Samples were left air-dried for at least 15 min at room temperature before the study. To classify the samples and determine the phase of the cycle they belong to, the protocol designed by Professor Odeblad was followed [22]. For the study, a 10 × 10 grid in the microscope eyepiece was necessary. Ten fields of the same sample were arbitrary chosen, and the CM present in every piece of the 10 × 10 grid of each field was observed. The percentages of each type of mucus were calculated by adding up the number of grid pieces containing each mucus type and dividing the result by the total number of grid pieces. With the sum of percentages, and the date of the last menstrual period (LMP), the final percentage of each type of mucus in a sample was obtained. This semiquantitative study allowed us to calculate the percentages of the different mucus types in each sample and also to determine the day of the cycle to which the sample belonged, using the model described by Professor Odeblad in 1994. The samples were classified as follows: early estrogenic (day −12 to day −6), late estrogenic (day −5 to −3), early ovulatory (day −2 to day 0), and late ovulatory (day +1 to +2), considering day 0 as the day of ovulation [57].

For the proteomic analysis, the remaining part of the CM sample was placed in plastic containers and mixed up in a 1:1 (*v/v*) ratio with a solution of aqueous trifluoroacetic acid (TFA 0.2% *v/v,* Mallinckrodt Baker B.V. Deventer, The Netherlands), centrifuged afterwards for ten minutes at 9200× *g*, and kept at −80 °C until analysis. A fraction of 0.5 mg of total protein (determined by the Bradford assay) of every CM sample was mixed with pH 8.0, 1 M ammonium bicarbonate (AMBIC, Sigma-Aldrich, St. Louis, MO, USA) reduced with 200 mM D,L-dithiothreitol (DTT, 10 mM final concentration, Sigma-Aldrich, St. Louis, MO, USA) (first for five minutes at 100 °C and for fifteen minutes at 50 °C afterwards), and alkylated with 200 mM iodoacetamide (IAA, 55 mM final concentration, Sigma-Aldrich, St. Louis, MO, USA) at room temperature in the dark for sixty minutes. Finally, 100 mM ammonium bicarbonate (pH 8) with trypsin (ratio 1:50 trypsin:protein) was added to the samples to let them digest overnight at 37 °C (Trypsin Gold MS Grade, Promega, Madison, WI, USA). The samples were acidified with an aqueous solution of TFA (0.2% *v/v*) to stop the digestion, and forthwith lyophilized and frozen. The samples were resuspended in 50 µL of aqueous formic acid solution (FA, 0.1% *v/v*, Merck, Darmstadt, Germany), grouped into three pools according to the menstrual phase, and analyzed in triplicate with a UHPLC-nanoESI Orbitrap Elite mass spectrometer (Thermo Fisher Scientific, Waltham, MA, USA). Chromatographic separations were performed on a UHPLC Ultimate 3000 RSLCnano system (Dionex, Sunnyale, CA, USA) equipped with a PepMap C18 (2 µm particles, 100 Å pore size) EASY-Spray column (15 cm in length × 50 µm of internal diameter (ID)) (Thermo Fisher Scientific) coupled to Acclaim PepMap100 nano-trap cartridge (C18, 5 µm, 100 Å, 300 µm i.d. × 5 mm) (Thermo Fisher Scientific). Separations were performed at 40 °C in gradient elution using 0.1% FA as eluent A, and an ACN/FA solution (99.9:0.1, *v/v*) as eluent B. The applied gradient was linear from 5 to 35% of solvent B in 120 min, at a flow rate of 0.3 µL min^−1^. The Orbitrap Elite instrument was operating in positive ionization mode at a 60,000 full scan resolution in 350–2000 *m/z* acquisition range, performing MS/MS fragmentation by collision-induced dissociation (CID, 35% normalized collision energy) of the 20 most intense signals of each MS spectrum in data-dependent scan (DDS) mode. MS/MS spectra acquisition was performed in the linear ion trap at a normal scan rate.

For tandem mass spectra analysis, the SEQUEST HT cluster, as a search engine opposite to UniProtKb/Swiss-Prot protein knowledgebase release protein database Homo Sapiens 2018-03, and the Thermo Proteome Discoverer (version 1.4.1.14) software were used. The following strict filtering was applied to the peptides in order to ensure a trustworthy identification: XCorr versus charge for doubly and triply charged ions, 1.8 and 2.5 respectively; both false discovery rate and high value peptide confidence less than 5%. Variable methionine oxidation; fixed carbamidomethylation of cysteines; oxidation of methionines; and phosphorylation of serine, threonine, and tyrosine as variable modifications were all searched for in the data. In order to analyze a proteic pattern for the CM for this investigation, the panel of proteins identified in at least one sample for each menstrual day of the cycle analyzed with an X score generated by the program greater than 1.5 was taken into consideration. The freely accessible PANTHER (protein annotation through evolutionary relationship) program (http://www.pantherdb.org/, accessed on 17 August 2020) was used to evaluate the proteins recognized by SEQUEST HT. The GO annotations for molecular function in the common proteins were assessed for the purposes of this study. For a p less than 0.05, statistical significance was established. During the bioinformatic analysis and common proteins, label-free quantification was performed using peak area calculation quantification by using the software Proteome Discoverer. The relative amounts of each peptide in a sample were estimated using this quantification methodology. During processing, the Proteome Discoverer application calculates peptide areas and uses them to compute protein areas for the proteins in the report automatically. The area of any given protein was calculated as the average of the three most prevalent unique peptides. Mini- Tab 1.7 software (Italy, GMSL, Nerbiano) was used to perform the statistical analysis. The cut-off points for upregulated proteins ≥1.5 and for downregulated proteins ≤0.667 were considered significant [58]. The samples were divided into 3 groups for analysis. The grouping criterion was the day of the menstrual cycle on which they had been obtained. Thus, the 6 samples obtained on day 7 of the cycle were analyzed together in a group, as well as the 6 samples from day 12 in another group, and the 5 samples from day 18 in a third group. First, the proteomic analysis of the three groups was carried out, and, later, the data analysis. The sample that was in the gestagenic phase was excluded from the proteomic study.

The study design was approved by the Ethical Commission on Animal and Human Experimentation (CEEAH) of the Autonomous University of Barcelona, CEEAH Code: 3250.

## 3. Results

### 3.1. Light Microscope

The observation of the samples by light microscopy allowed the identification of the various types of CM according to their crystallization. Samples were classified according to the phase of the menstrual cycle to which they belonged.

#### 3.1.1. Identification and Description of the Different Types of CM

The crystallographic pattern of each type of secretion made it possible to identify the following types of CM: L, S, P, and G, with their different subtypes as described below:

- L mucus

L mucus presented with crystals forming a central axis, from which branches emerged perpendicular to the central axis at a 90° angle. Those branches may have one or more secondary branches, perpendicular to each other. The global appearance of this mucus is what has generally been defined as “fern morphology”.

L mucus is secreted from several days before ovulation to ovulation, and has the function of filtering the sperm, constituting a very precise natural selection. The diameter of the pores makes it difficult for sperm to advance, although not entirely, so only the best sperm can pass this filter. Its production is stimulated by moderate levels of estrogen [57].

- S mucus

S-type mucus crystallized in thin parallel rows and presented 3 subtypes: S1 mucus was the simplest type of S mucus observed. It consisted of more or less polygonal crystals that were arranged in a row. The crystals were not linked to each other, but their arrangement suggested a linearity between them. S2 mucus arranged forming sets of rows parallel to each other. With higher magnification, the row of crystals presented few discontinuities. In S3 mucus, the crystals were observed in rows with short branches. These branches may or may not have other branches or secondary branches. The length of the axis of the branches did not exceed the length of the central axis.

S mucus is secreted around ovulation. It constitutes the great highways through which sperm can swim once they have been properly filtered by type L. It requires high concentrations of estrogen to be secreted. Norepinephrine appears to also be a biological stimulus for mucus S [57].

- P mucus

P-type mucus had a crystalline morphology consisting of a central axis and branches that form 60° angles with this axis.

Five subtypes of P mucus could be observed: P6B, P2, Pa, P4, and Pt.

P6B mucus presented a hexagonal symmetry, with six well-defined axes. The morphology was stellate. Branches departed from the axes at an angle of about 60°.

P mucus has the function of filtration and final sugar supply [57].

- G mucus

G-type mucus is characterized by having a high density of crystals, which appeared as loose crystals or together, creating amorphous clusters. In some cases, preparations with G mucus had a high cellular content (epithelial cells of the cervix, leukocytes, and lymphocytes).

Type-G mucus forms a plug in the cervix that closes it, making it impenetrable to sperm, and defending the genital tract from infection. G mucus is present in the phases of infertility [57].

**Figure 1:**
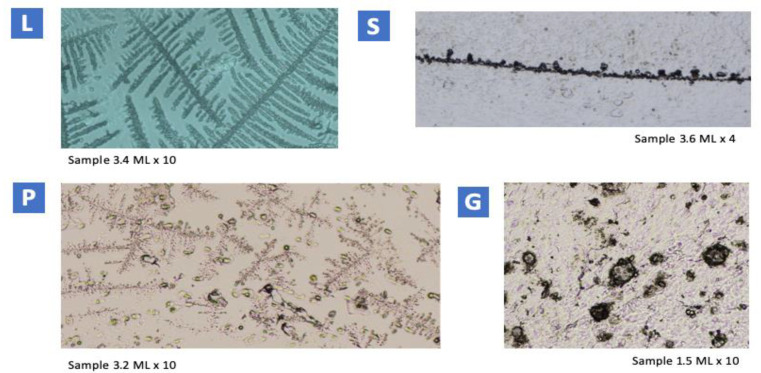
Examples from each CM type. L mucus: fern-like figure. S mucus: more or less circular crystals, close to each other, arranged in a row with no branching. P6B mucus: in the central part of the image, hexagonal symmetry and six asymmetric axes. Branches form a 60° angle with the axis. G mucus: loose crystals of miscellaneous morphology can be seen.

An example of each CM type is shown in Figure 1.

#### 3.1.2. Classification of the Samples According to the Phase of the Cycle

Samples were classified according to the protocol designed by Professor Odeblad [22]. The most relevant data of the analyzed samples are shown in Table 1.

### 3.2. Proteomic Analysis

The proteomic analysis of the 17 samples was carried out by dividing them into three groups according to the day on which the sample was collected. (1) Samples from day 7: the first group included six samples, all of them in the early estrogenic phase. (2) Day 12: the second group included six samples, most of them in the early estrogenic phase. (3) Day 18: the third group included five samples in the ovulatory phase.

#### 3.2.1. Proteins Identified

The proteomic analysis of the samples of the three groups together allowed the identification of a total of 48 proteins. The complete list of proteins is reported in Table 2. The following information is reported for each protein: UniPROT Code, gene name, molecular weight in kDa, isoelectric point, and description.

Among the 48 proteins identified and listed in Table 2, there were: immunoglobulin chains (IGHA1, IGHG1, IGHG2, IGHG3, IGKC, JCHAIN, IGLC1), neutrophil defensin 1 (DEFA1), WAP four-disulfide core domain protein 2 (WFDC2), serine protease inhibitor Kazal-type 5 (SPINK5), and lactotransferrin (LTF), all implicated in immunity.

#### 3.2.2. Identification of Peaking Proteins and the Biological Processes in Which They Participate

The peak search algorithm allowed the mass spectrometry data to be identified and a list of peaks and relative abundances to be generated. The peaks represent the peptide fragments for a given mass and charge. Eleven peaking proteins were identified in the global analysis of the samples. The biological processes in which the peak proteins participate are the following:

- Small proline-rich protein 3 (SPRR3), cornifin-B (SPRR1B), keratin, and type II cytoskeletal 6A (KRT6A) are involved in keratinization;
- Cystatin-B (CSTB) and serine protease inhibitor Kazal-type 5 (SPINK5) are protease inhibitors;
- Heat shock protein beta-1 (HSPB1) participates in the biological process of host–virus interaction;
- Immunoglobulin kappa constant (IGKC), immunoglobulin heavy constant gamma 1 (IGHG1), polymeric immunoglobulin receptor (PIGR), and protein S100-A8 (S100A8) participate in immunity;
- Serotransferrin (TF) participates in transport.

#### 3.2.3. Bioinformatic Analysis of Molecular Functions

Bioinformatic analysis of the molecular functions of the proteins revealed that most of the CM constituents on day 7, when all samples were in the early estrogenic phase, were binding proteins. The second largest group were proteins that have catalytic activity, followed by proteins that regulate molecular function, and transporter and structural activity.

The bioinformatic analysis of the molecular functions of the proteins of the samples from day 12 also reflected that the majority group were binding proteins and the second largest group were proteins with catalytic activity. The percentages were practically identical to those in day 7.

The comparative analysis of the GO annotations for the molecular function of the proteins identified on day 18, when most of the samples were in the ovulatory phase, revealed an increase in catalytic activity compared to the previous phases, although binding activity remained predominant.

#### 3.2.4. Variability in Protein Abundance

A detailed study was carried out to analyze possible significant changes in the abundance of the proteins between the estrogenic phase and the ovulatory phase. To do so, samples from days 7 and 12 were analyzed together and compared with the samples on day 18.

**Figure 2:**
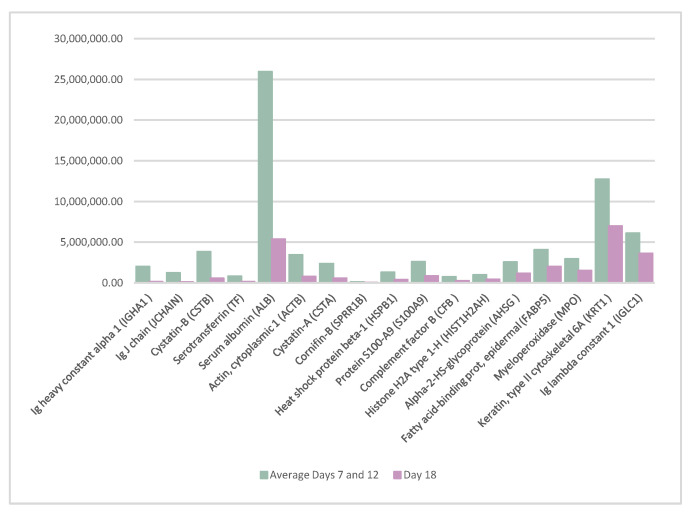
Proteins whose abundance decreased significantly on day 18 of the cycle when compared to the average of days 7 and 12 of the cycle.

Seventeen proteins significantly decreased their abundance on day 18 of the cycle compared to days 7 and 12. The list of the proteins is as follows: Ig heavy constant alpha 1 (IGHA1), Ig J chain (JCHAIN), cystatin-B (CSTB), serotransferrin (TF), serum albumin (ALB), actin cytoplasmic 1 (ACTB), cystatin-A (CSTA), cornifin-B (SPRR1B), heat shock protein beta-1 (HSPB1), protein S100-A9 (S100A9), complement factor B (CFB), histone H2A type 1-H (HIST1H2AH), alpha-2-HS-glycoprotein (AHSG), fatty acid-binding prot, epidermal (FABP5), myeloperoxidase (MPO), keratin, type II cytoskeletal 6A (KRT1), and Ig lambda constant 1 (IGLC1). The difference in the abundance of these proteins is shown in Figure 2.

**Figure 3:**
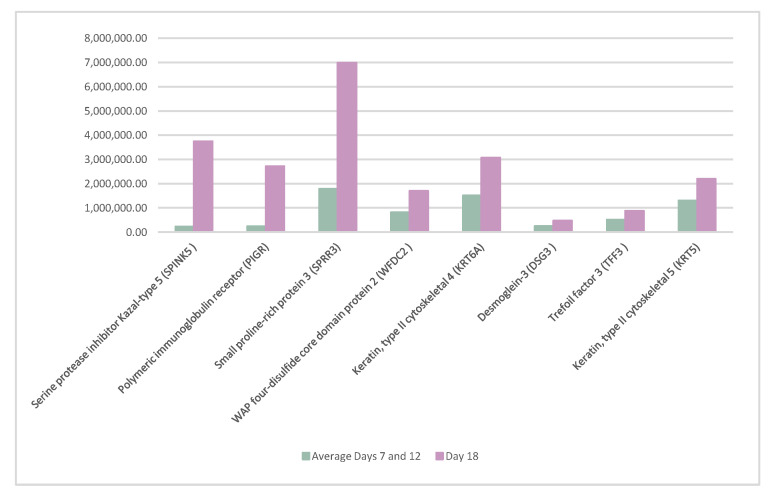
Proteins whose abundance increased significantly on day 18 of the cycle when compared to days 7 and 12 of the cycle.

Furthermore, eight proteins, serine protease inhibitor Kazal-type 5 (SPINK5), polymeric immunoglobulin receptor (PIGR), small proline-rich protein 3 (SPRR3), WAP four-disulfide core domain protein 2 (WFDC2), keratin type II cytoskeletal 4 (KRT6A), desmoglein-3 (DSG3), trefoil factor 3 (TFF3), and keratin type II cytoskeletal 5 (KRT5), significantly increased their presence on day 18 of the cycle. The details of these proteins are shown in Figure 3.

It is possible to determine the area of any peptide by performing the quantification of the peak area calculation. This quantification method is useful for knowing the relative amounts of all peptides in a sample. The most abundant proteins on days 7, 12, and 18 of the cycle were identified. Absent proteins in each day were also identified. On day 18 of the cycle, seven proteins were absent. Table 3 reveals the list of the absent proteins on this day, when most of the samples were in the ovulatory phase.

All the absent proteins listed in Table 3 are involved in the immune response.

## 4. Discussion

Several investigations have focused on the search for a pre-ovulatory biochemical parameter that could predict ovulation in advance. This discovery would allow to define, with greater accuracy, the combined fertile window.

With this aim, the present study correlates, for the first time, the analysis of the crystallographic patterns and the proteomic analysis of CM samples along the menstrual cycle.

At a biophysical level, the identification with light microscopy of the four main types of CM crystallographic patterns correlated with that previously described in the literature [23,24]. The percentages obtained from the different types of CM are similar to those found in previous studies [24]. The methodology designed by Professor Odeblad [22] was simple to implement and enabled an accurate classification of the samples according to the phase of the cycle to which each sample belonged. Cyclical changes in mucus crystallization along the cycle have been evidenced and were consistent with earlier investigations [22,23,24]. Since there are a limited number of studies in this field, these findings are interesting, as they support the previous ones.

Our results suggest that ovulation happens later in the menstrual cycle than commonly assumed by the clinical guidelines. Our findings are consistent with recent works that outlined that ovulation occurred at around day 18 of the cycle [10].

Even if the proteomic composition of CM has been studied previously, the number of proteins that it contains, or how exactly the CM proteome undergoes cyclical changes along the cycle, are still unknown [56].

In 2015, Grande et al. analyzed, for the first time, the modifications in the CM proteome during the phases of the menstrual cycle, using high-resolution mass spectrometry, implemented by quantitative tools. The proteomic approach revealed differences in the expression of various CM proteins during the menstrual cycle, which are involved in inflammation, structural activity, and the defense system [56].

The proteomic analysis of the samples of the present study has allowed the identification of 48 proteins. Twenty-one out of the forty-eight proteins identified in this research coincide with the thirty-eight described as constitutive proteins of CM in the study carried out by Grande et al. This variability may be due to the normal inter- and intra-individual variability that may exist in healthy women [56]. Furthermore, a different methodological approach has been used in this study, since we performed proteomic analysis on pools of CM samples and not on single samples. Additionally, it may also be due to the use of a different sensitive technical instrument, such as a nano-ESI source, compared with the ESI source adopted in the previous study. Finally, more updated bioinformatic databases and software have been used for bioinformatic analysis.

Bioinformatic analysis for the molecular functions in both the previous study of Grande et al. and the current one revealed that most of the CM constituents were binding proteins and proteins with catalytic activity.

Among the 48 proteins identified, it is worth highlighting the presence of immunoglobulin chains (IGHA1, IGHG1, IGHG2, IGHG3, IGKC, JCHAIN, IGLC1), neutrophil defensin 1 (DEFA1), WAP four-disulfide core domain protein 2 (WFDC2), serine protease inhibitor Kazal-type 5 (SPINK5), and lactotransferrin (LTF), which suggest the importance of the defensive role of CM, a finding that coincides with previous studies [56,59,60,61,62,63].

This study allowed the identification of 11 peaking proteins. Small proline-rich protein 3 (SPRR3), which is involved in the keratinization process, was present among the constitutive proteins of CM described by Grande et al. [56].

Seven out of the seventeen proteins that significantly decreased their abundance in the ovulatory phase were identified as constitutive proteins of the CM by Dr. Grande et al. as listed: Ig heavy constant alpha 1 (IGHA1), Ig J chain (JCHAIN), serotransferrin (TF), serum albumin (ALB), actin cytoplasmic 1 (ACTB), protein S100-A9 (S100A9), and myeloperoxidase (MPO). Three out of the eight proteins that significantly increased their presence on day 18 of the cycle were also identified previously by Grande et al.: small proline-rich protein 3 (SPRR3) and WAP four-disulfide core domain protein 2 (WFDC2) as constitutive proteins, and desmoglein-3 (DSG3) as an ovulatory-specific protein.

Twenty-five proteins have been identified in the present study as potential biomarkers to predict ovulation. This is a promising finding, as anticipating ovulation is a key element for the increasing number of couples interested in identifying the fertile window for the purpose of conceiving.

The fact that only 40 proteins out the total 48 were present on day 18 of the cycle confirm the results of previous studies, where most of the enzymes described in the CM show a cyclic pattern, with a decrease in the number of proteins from 3 to 5 days prior to ovulation [29]. Chemical modifications of the CM during ovulation include the decrease of certain soluble proteins [30].

It is also very remarkable that these seven absent proteins on day 18 of the cycle are involved in the immune response; that is, on day 18 of the cycle, when most of the samples were in the ovulatory phase, the abundance of immunoglobulins and other elements involved in the defense of the body turn out to be lower than on days 7 and 12. In this sense, Odeblad stated that the highest content of immunoglobulins and defenses, in general, against infections is found in mucus G, present in an almost absolute percentage in the infertile phases of the cycle [23].

These determinations could be the answer to the question of why the immune system does not react by destroying the sperm, as it is foreign to the body; that is, the coordinated changes that occur in the biochemical composition of the CM around the time of ovulation specifically allow the entry of a foreign body, such as the sperm.

The CM blocks foreign organisms from entering the uterus. However, this blockade is selective and allows access to sperm at certain phases of the cycle.

Moreover, the spermatozoa, which, by themselves, would die in hours thanks to the changes in the protein composition of the CM around the ovulatory phase, survive, accommodated in the cervical crypts for up to 5 days, ready to go towards the most distal part of the fallopian tube when ovulation happens [5].

In the literature, it has been suggested that CM is a reliable predictor of fertility [64], and our study may provide a basis for a better anticipated identification of ovulation.

To our knowledge, there is no previous study in the literature that correlates the analysis of light microscopy and proteomic analysis in CM samples.

We must also acknowledge certain limitations of our experimental design, as we performed our study on a relatively small sample scale.

CM is an excellent source of protein biomarkers. The cyclical variations of the cervical proteins, due to hormonal variations, make them potential reliable biomarkers of ovulation.

Continuing to deepen the knowledge of CM and accurately defining its protein composition, as well as its variations throughout the cycle and the variability between healthy women and women with different gynecological pathologies, are key factors in the field of reproductive and gynecological health.

## 5. Conclusions

In this investigation, cyclical changes in the composition of the CM during the menstrual cycle have been evidenced both at the biophysical and biochemical level.

Our study provides further support to the insights of the evolution of the different CM crystallographic patterns in the menstrual cycle when observed by light microscopy.

The proteomic analysis enabled the identification of 48 proteins whose abundance varied along the menstrual cycle.

In our research, 25 proteins are proposed as potential preovulatory biochemical parameters to prospectively identify ovulation and, consequently, to predict the combined fertility window.

The findings of the study point towards the idea that the coordinated changes in the composition of the CM around the time of ovulation are addressed at allowing the entry of a foreign body, such as the sperm.

## Figures and Tables

**Table 1:** Most relevant data from the samples analyzed using light microscopy.

Age	ID Number of the Sample (Day. Volunteer ID Number)	Percentage of the Different CM Types in Each Sample	Phase of the Menstrual Cycle
28	1.1	100% G	Early Estrogenic
	2.1	100% G	Early Estrogenic
	3.1	100% G+	Gestagenic
25	1.2	100% G	Early Estrogenic
	2.2	50% G 50% L	Late estrogenic
	3.2	85% G 10% L. 5% P	Late Ovulatory
27	1.3	100% G	Early Estrogenic
	2.3	100% G	Early Estrogenic
	3.3	100% G	Early Estrogenic
30	1.4	100% G	Early Estrogenic
	2.4	100% G	Early Estrogenic
	3.4	80% L 16% S 4% P	Early Ovulatory
29	1.5	100% G	Early Estrogenic
	2.5	90% G 10% L	Early Estrogenic
	3.5	23% G 50% L 15% S 2% P	Early Ovulatory
28	1.6	100% G	Early Estrogenic
	2.6	100% G	Early Estrogenic
	3.6	80% L 17% S 3% P	Early Ovulatory

**Table 2:** List of the 48 proteins identified in the proteomic analysis.

UniPROT	Gene Name	Molecular Weigth [kDa]	Isoelectric Point	Description
P04217	A1BG	13.2	6.13	Alpha-1B-glycoprotein
P01859	IGHG2	69.3	6.28	Immunoglobulin heavy constant gamma 2
P01860	IGHG3	18.1	8.57	Immunoglobulin heavy constant gamma 3
P01857	IGHG1	14.3	8.75	Immunoglobulin heavy constant gamma 1
P08311	CTSG	37.6	6.51	Cathepsin G
P02533	KRT14	11.0	5.50	Keratin, type I cytoskeletal 14
P05109	S100A8	16.5	9.16	Protein S100-A8
P01834	IGKC	15.2	8.68	Immunoglobulin kappa constant
P22528	SPRR1B	15.2	7.01	Cornifin-B
P01591	JCHAIN	22.8	6.40	Immunoglobulin J chain
P01876	IGHA1	16.0	7.28	Immunoglobulin heavy constant alpha 1
P02787	TF	53.5	6.10	Serotransferrin
B3EWG3	FAM25A	8.6	5.92	Protein FAM25A
P00751	CFB	13.9	10.32	Complement factor B
P61626	LYZ	11.1	7.56	Lysozyme C
P02511	CRYAB	9.9	8.48	Alpha-crystallin B chain
P35321	SPRR1A	9.9	8.48	Cornifin-A
P04792	HSPB1	11.8	6.52	Heat shock protein beta-1
Q96KK5	HIST1H2AH	10.2	6.99	Histone H2A type 1-H
P13646	KRT13	10.8	7.03	Keratin, type I cytoskeletal 13
P32926	DSG3	11.3	7.87	Desmoglein-3
P04080	CSTB	78.1	8.12	Cystatin-B
P01040	CSTA	13.0	4.84	Cystatin-A
P60709	ACTB	9.3	6.15	Actin, cytoplasmic 1
P06702	S100A9	12.3	8.82	Protein S100-A9
Q07654	TFF3	77.0	7.12	Trefoil factor 3
P02765	AHSG	41.3	7.90	Alpha-2-HS-glycoprotein
P04083	ANXA1	66.0	8.12	Annexin A1
P02788	LTF	60.0	8.00	Lactotransferrin
P05164	MPO	36.1	8.19	Myeloperoxidase
P04264	KRT1	41.7	5.48	Keratin, type II cytoskeletal 1
Q14508	WFDC2	28.8	11.19	WAP four-disulfide core domain protein 2
P19957	PI3	62.3	7.74	Elafin
Q01469	FABP5	35.9	7.59	Fatty acid-binding protein, epidermal
P13647	KRT5	20.1	7.33	Keratin, type II cytoskeletal 5
P01833	PIGR	83.8	8.97	Polymeric immunoglobulin receptor
P19013	KRT4	39.3	5.72	Keratin, type II cytoskeletal 4
P69905	HBA1	51.5	5.16	Hemoglobin subunit alpha
P0CG04	IGLC1	18.1	5.24	Immunoglobulin lambda constant 1
Q9UBG3	CRNN	49.6	4.96	Cornulin
Q9NQ38	SPINK5	13.9	10.89	Serine protease inhibitor Kazal-type 5
P03973	SLPI	57.2	6.61	Antileukoproteinase
P68871	HBB	54.2	5.86	Hemoglobin subunit beta
P23527	HIST1H2BO	120.6	8.06	Histone H2B type 1-O
P02768	ALB	83.2	5.74	Serum albumin
P02538	KRT6A	38.7	7.02	Keratin, type II cytoskeletal 6A
Q9UBC9	SPRR3	85.5	7.06	Small proline-rich protein 3
P59665	DEFA1	107.5	5.00	Neutrophil defensin 1

**Table 3:** List of the absent proteins on day 18 of the cycle.

UniPROT	Gene Name	Description
P01834	IGKC	Immunoglobulin kappa constant
P01857	IGHG1	Immunoglobulin heavy constant gamma 1
P01859	IGHG2	Immunoglobulin heavy constant gamma 2
P01860	IGHG3	Immunoglobulin heavy constant gamma 3
P08311	CTSG	Cathepsin G
P05109	S100A8	Protein S100-A8
P04217	A1BG	Alpha-1B-glycoprotein

## Data Availability

The data presented in this study are available on request from the corresponding author.
